# On Female Diseases

**Published:** 1829-10-01

**Authors:** 


### Medico-Chirurgical Review,

No. XXII.

JULY 1, to OCTOBER 1, 1829.

### I

### ON FEMALE DISEASES.

1. Commentaries on Some of the More Important of the Diseases of Females. By *Marshall Hall*, M.D. &c. 1827.
2. An Account of Some of the Most Important Diseases peculiar to Women. By *Robert Gooch*, M.D. 1829.

[Art I.]

The appearance of Dr. Gooch's work reminded us of a promise made in October 1827, and not yet fulfilled. We then reviewed the *first* part of Dr. Hall's book, and announced our intention of dedicating an article to the second part. We are not sorry that some delay has taken place, as it will enable us not only to exhibit a careful analysis, but to institute a parallel between the works of two highly talented physicians.

In deference to seniority, and because it will best suit our plan, we shall commence this article with the second part of Dr. Hall's book. We may premise, however, that Dr. Hall's observations relate to those puerperal diseases which are *sporadic* :—those of Dr. Gooch, chiefly to the epidemic form of these diseases ; except indeed what relates to the subject of puerperal insanity. This is the *first* distinction to be made in the investigation of the class of puerperal diseases ; for however similar these two parts of the class may be, they are so greatly modified as to be of very different prognosis, the sporadic being far less fatal than the epidemic.

Dr. Hall first treats of puerperal diseases in general, tracing their various causes, and the various co-operation of these causes, in their production.

Puerperal diseases "may be divided into those which occur in the earlier, and in the latter periods of pregnancy,—immediately before, and after, and during the act of parturition,—during what is termed the puerperal state,—and during the period of lactation."

In the early period of pregnancy many organs suffer, in consequence of the source of irritation then set up in the system. In the latter periods, general causes combine their influences to endanger the state of the brain ; these are chiefly, uterine and intestinal irritation, concurring with the actual pressure of the gravid uterus upon the various viscera and vessels situated behind it, and the state of plethora of the vascular system occasioned by this pressure. During parturition the contractile efforts of the uterus and of the abdominal muscles, add another source of danger to those already mentioned. Several sources of danger are removed when delivery has taken place ; and yet this is not always sufficient to protect the patient from an attack of convulsion. Such an attack may occur after delivery, or even although the system be in a state of exhaustion from hæmorrhage, a state which is not incompatible with congestion of the brain. This last point is particularly insisted upon by our author, and so it is, as we shall have to state presently, by Dr. Gooch. Immediately after delivery the emptied condition of the uterus and abdomen may co-operate with loss of blood in inducing a state of inanition or exhaustion. To these sources of puerperal diseases, obtaining immediately after delivery, must also be added another not less important, viz. the effects of protracted suffering, of violent pain, of mental alarm, and of the "shock" of parturition. The next series of morbid affections are those more generally esteemed puerperal, and do not occur until some hours after delivery. They are intestinal irritation, the effects of loss of blood, and uterine or peritoneal inflammation. There are also two sources of irritation in the condition of the mammæ and of the uterus. Dr. Hall has particularly insisted upon "mixed cases," or cases which result from the co-operation of two or more of these causes of puerperal diseases; an important point. Lastly, there are the various forms of epidemic puerperal disease. After puerperal disease, properly so called, an interesting object of inquiry presents itself in the effects of undue lactation. These are treated of at length in the third part of Dr. Hall's work, though we think they had better have formed the concluding part of this. The effects of undue lactation have not been so fully discussed before; and probably Dr. Hall was well fitted for treating this subject on account of his well-known attention to the effects of loss of blood, for it is only another form of exhaustion. The previous state of the health of the patient is also important to be considered in estimating the precise nature and character of puerperal disease.

From these views it is plain that there are several distinct cases of those puerperal diseases which occur sporadically. These are inflammation, intestinal irritation, and exhaustion from loss of blood; besides which, there are "mixed cases" which combine two or more of these. And all are apt to be further modified by the circumstances of uterine or mammary irritation. It is equally obvious that the treatment of different puerperal cases must be very different, and that, in those which prove fatal, the morbid appearances must not only be very different, but in some absent altogether. If, as we suspect, under the common designation of puerperal fever, all these various forms of disease have frequently been comprehended, we cannot feel surprised that discrepancy of opinion in regard to their treatment should have so long prevailed.

The first part of Dr. Gooch's work treats of *epidemic* puerperal diseases. It is well known how completely and how dreadfully the character of these affections is modified by this circumstance of their epidemic prevalency. Yet it is singular that very similar differences obtain in the epidemic and in the sporadic forms:—some recover under the prompt and efficient use of the lancet; others do not bear blood-letting;—some present all the traces of inflammatory action, on inspection after death; others leave no morbid appearances;—as might be expected, the most successful modes of treatment are equally dissimilar. These points are alike insisted upon by both the authors before us; as one treats of the sporadic and the other of the epidemic disease.

In the sporadic puerperal peritonitis the pathognomonic symptom is tenderness of the abdomen, increased, or perhaps only discovered on pressure; this pain, besides varying in degree, may be partial or general. There is frequently rigor, but not always, and perhaps not of the severest kind; the skin is not always hot, nor the pulse extremely frequent; there is not necessarily pain of the head. The chief object of attention is the state of the abdomen. The patient does not easily faint if placed upright and bled. The disease either subsides or passes into the sinking state.

This affection is too well known to need to be exemplified, in this place, by cases.

In intestinal irritation there may be equal, nay, severer pain and tenderness of the abdomen; severer rigor; greater heat of the surface; and more intense pain, perhaps with throbbing of the head, and even intolerance of light and noise. This disease is, indeed, frequently marked by more urgent symptoms than inflammation. There is greater susceptibility to fainting on taking blood; the stomach or the bowels are more obviously disordered; and the symptoms are relieved by emetic, aperient, and opiate remedies. Dr. Hall observes, in regard to this disease—

"I have scarcely had an opportunity of examining the state of the internal organs after death; for in general the patients affected by intestinal irritation have recovered. But I have no doubt that such an examination would illustrate the following important remark of the late Dr. Denman;—'We have been told, that in the dissection of some who are said to have died of puerperal fever, no appearances of inflammation have been discovered; but I should suspect that, in such cases, some important appearances had been overlooked, or that errors had been committed as to the nature of the disease, and probably in its treatment.'

"A due consideration of the effects of intestinal irritation will also serve to elucidate other cases of morbid affection, in which the appearances of inflammation were looked for on dissection, but were not found. This observation applies particularly to affections of the head, heart, and abdomen.

"In several cases of this morbid affection, which I had the opportunity of examining many years ago, no morbid appearances were found on the most careful inspection." 201.

The subject is illustrated by numerous cases. Of these some plainly depended upon indigestible substances taken; in others upon the state of the bowels; in the former case the head is chiefly affected, in the latter both the head and abdomen, together or separately, but chiefly the latter. We shall select a case or two in illustration.

"Mrs. ——, aged 35, continued well for several days after delivery, until she partook of some ham; she soon began to complain of pain of the head, and vertigo; on going to bed the pain and vertigo increased, and she became affected with rambling and starting, with great intolerance of light, so that she complained bitterly on a candle being brought into the room, and with equal intolerance of noise and disturbance. The pain of the head occupied the occiput principally; there was also pain in the region of the stomach, and general soreness over the abdomen.

"The intelligent surgeon who attended this patient prescribed a purgative enema, followed by a pill consisting of five grains of calomel and one of opium, and an active purgative mixture,—and directed the feet to be fomented. The following morning every symptom had disappeared. The patient reported that the action of the purgative and the fomentation had promptly relieved her. She added an expression of surprise at having obtained such immediate relief, having on a former occasion experienced a similar attack and been bled to no purpose, as she had continued to suffer for many days." 202.

"The following case, which I extract from the interesting paper of Dr. John Clarke,* to which I have already referred, p. 163, is still more extraordinary.

"'Mrs. T. came to London expressly for the purpose of lying-in. She was a healthy woman, the mother of several children, and had always passed through the period of her confinement without any unfavourable complaints.

"'For the purpose of her confinement, she resided in a furnished house, where two streets crossed each other, and there was a mews at the back of the house. Here she lived for three weeks before her labour. She had a very natural delivery, and slept well afterwards. By the end of 10 or 12 days, she was well, and free from any disorder.

"'In the course of one night she was seized with a severe pain in her head, attended with considerable impatience of light. These symptoms became more violent towards the morning, so as to excite great alarm in her husband, who immediately came to the writer. On learning that she had been perfectly well on the preceding day, he asked if she could attribute the pain to any cause. She replied that she knew of none, unless that, from the situation of the house, she heard every carriage which passed the streets, and every carriage which entered or left the mews. But as she had been in the house five weeks without having found any inconvenience from it before, this did not appear a probable way of accounting for it.†

"'Every inquiry respecting her diet was made, and it appeared that she had eaten nothing but the most simple food. The writer, upon receiving this information, observed, that he was glad that she had eaten no oysters. To this observation she replied, that she had, two days preceding the attack, eaten oyster-sauce to some boiled chicken, but that she could not comprehend how that should produce such a violent pain in the head; and she appeared anxious to know, whence the satisfactory conclusion was drawn from her having before said, that she had eaten nothing but simple food, having forgotten the oysters, of which she had swallowed about a dozen. An answer to her inquiry on this head was avoided.'

"I would earnestly recommend the whole of this essay to the reader's attentive perusal. It is quite obvious that the symptoms which are detailed in it, as resulting from partaking of oysters in the puerperal state, may originate from any other equivalent source of irritation of the stomach." 204.

We suspect that more puerperal cases are induced by improper articles of food given or taken, than is generally supposed. We are induced to press this fact upon the attention of our readers from its exceeding importance, especially in regard to the cure. In every case of puerperal disease, the strictest and minutest inquiries should be made into the diet; and in every case, in which some improper or even doubtful article of food has been taken, a dose of ipecacuanha should be administered instantaneously. In half a dozen hours after such an indiscretion we have known patients affected with sickness, fainting, pain, head-ache, delirium, and a pulse of 140 or 150, and life placed in the utmost jeopardy!

We now select a case or two dependent upon the state of the bowels. In such cases the abdomen is most apt to be affected; but the head may suffer from pain, throbbing, and intolerance of light and noise, and even delirium; hence the common circumstances of tied up knockers, darkened rooms, &c., and, perhaps, even the common phrase of "in the straw." The attack of

* "Transactions of the College of Physicians, vol. v. pp. 125, 126."

† "It is plain that this circumstance was the effect, and not a cause, of the disease, and consisted in intolerance of sound so common in these cases." M. H.

puerperal affection dependent on intestinal irritation, is frequently induced by the action of a purge. This is a well-known and important fact. And it would suggest the propriety of washing out the large intestine by a copious injection of some bland mucilaginous fluid, after delivery, in preference to the too customary plan of administering a purge.

"Mrs. ———, a healthy young person, was confined on January the 20th, 1820. On the preceding day she had experienced inefficient wearying pain. On the morning of her confinement the pains were strong, but the os uteri was found to be rigid; she was therefore bled to eighteen ounces, and her labour was soon afterwards completed.

"Mrs. ———, continued well until the succeeding morning, when she was affected with severe shivering, which was repeated three times, occupying about the space of three hours. The rigors were succeeded by great heat of the skin, and by great sickness, retching, and vomiting. An enema and purgative medicine were administered; much hardened fæces were expelled, together with a fluid having the appearance of yolk of egg; and much relief was experienced. In the evening, and during the night, however, there was great heat of surface; there were much restlessness and constant changes of posture, and throwing the arms about and out of bed; the sleep was disturbed by startings and slight delirium; there were head-ach, confused vision, and much humming noise; and there was great faintishness on any attempt to assume the erect posture. She was directed to take the effervescing mixture.

"On the morning of the 22d the sickness returned; the purgative medicine had acted; there was considerable uterine discharge. A draught was given with thirty-five drops of the tinctura opii.

"I saw the patient about one o'clock: the pulse was then 144; there were head-ach, intolerance of light, dimness and imperfection of the vision, and great humming noise in the ears; there was some beating of the carotids visible externally; there were restlessness, changes of posture, throwing the arms out of bed, faintishness if raised to the erect position, a feeling of want of air, and relief on smelling vinegar. A draught with thirty-five drops of the tinctura opii and a dram of the spiritus ammoniæ aromaticus, was ordered to be taken immediately, and to be repeated in three hours; a lotion, consisting of a dram of sulphas zinci and a pint of water, was directed to be applied to the pubes, and within the vagina. An aperient draught was prescribed, but not given.

"In the evening the pulse was 130; there had been comfortable, refreshing, and undisturbed sleep; all the symptoms were abated; the bowels had been purged; the uterine discharge was diminished. A draught with ten drops of the tinctura opii and half a dram of the spiritus ammoniæ aromaticus, was prescribed to be taken every five hours; the effervescing medicine was continued; the face and hands are directed to be washed with a lotion when hotter than natural.

"Early on the morning of the 23d, there was an attack of troublesome coughing. At ten o'clock the symptoms were nearly as on the preceding evening; at night they were still further mitigated,—the pulse being 120, the bowels open, the uterine discharge more scanty.

"On the succeeding day, Mrs. ———, complained most of general stiffness and aching of the limbs, and the pulse was 125. The opening medicine was given, the opiate draughts were again prescribed, and the lotion was omitted. In the evening Mrs. ——— was relieved, and the pulse was 120.

"On the next day there was little complaint; the pulse was 108, the bowels open, and the lacteal discharge natural. All these symptoms at length subsided; but soon after this time, the vein which had been pierced in the arm began to inflame, and this new but terrible disease, proceeded, in spite of every remedy, and destroyed the patient." 208.

We now proceed shortly to detail the characters of that puerperal case which depends principally upon the effects of loss of blood. These seldom, rather never, exist alone. They are generally conjoined with some of the effects of intestinal irritation; and they modify almost every case of that disease; but they rarely shew themselves in conjunction with inflammation.

There is seldom rigor or excessive heat; but there may be transient chills, transient flushes. The countenance and especially the prolabia are pallid. The head, the carotids, and the heart throb; there is intolerance of sound; (intolerance of light belongs rather to intestinal irritation;) there is a characteristic tendency to syncope on assuming the erect posture, and on taking blood. Sometimes the case resembles an affection of the head, sometimes disease of the heart.

Into the investigation and description of the effects of loss of blood Dr. Hall has entered fully, too fully for us to follow him here; we are compelled, therefore, to refer to his work for complete information on this very important and interesting subject. We extract the following case, which, in the commencement, conjoined some effects of intestinal irritation, but afterwards consisted more purely of those of loss of blood.

*Case.* "Mrs. ——— aged 35, was confined on Friday the 11th of June. For several weeks previously to delivery, she had been subject to pain of the head, and of the left side, which were relieved by an attention to the state of the bowels.

"After the expulsion of the placenta, there was considerable hæmorrhagy, which induced great exhaustion; two doses of forty drops of tinctura opii were given within two hours, with the effect of producing sleep. The flow of milk commenced on the same day, and was very copious.

"About three hours after delivery, Mrs ——— was seized with a violent pain of the crown of the head, confined to a space which could be covered by the hand; the pulse was 80 only; there was much thirst; the tongue was little affected; the skin was natural. This pain was relieved by the cold lotion, and opening medicines, and Mrs. ——— continued better during ten days.

"On the night of Monday, June the 21st, Mrs. ——— was taken about 12 o'clock, with severe shivering, which was succeeded by intense heat and dryness of the skin, great pain of the head, and intolerance of light and of noise. At ten o'clock on the succeeding morning, these symptoms still continued; the pulse was from 120 to 130, and sharp; the pain of the head was throbbing, and the head felt as if bound tight; the tongue was parched. Ten ounces of blood were taken from the arm, which produced temporary faintness, but some relief; the cold lotion was applied to the temples. At seven o'clock in the evening, the pain of the head was as severe as ever, especially if the lotion were not constantly applied; the pulse was 120; the tongue not so dry; the blood already drawn was buffy. Twelve ounces of blood were taken from the arm. This was followed by great faintness, and gasping breathing—to such a degree, indeed, as to lead to the apprehension of dissolution even. On recovery, the pain, and intolerance of light and sound remained as before; the pulse rose to 130. Leeches were applied to the temples and the cold lotion over the head; two grains of calomel were ordered to be taken every two hours; and an opening mixture and an enema were prescribed.

"At four o'clock of the morning of Wednesday the 23d, the symptoms continued with little change; the pulse was 120; there was much gaping. Six leeches were applied to the temples, a blister to the nape of the neck, and the medicines were continued.

"On Thursday morning, the 24th, the pulse was 100, and she appeared better, but complained of a degree of beating of the heart. At four in the afternoon the pulse was 120, the breathing was deep, sighing, and rare, and there was a sense of fluttering at the heart, the affection of the head still continuing. Two grains of opium and five of calomel were ordered to be taken immediately.

"At two o'clock on Friday morning, Mrs. —— was distressed with a feeling of hurry, of impending dissolution, and of being 'overcome' by sleep; the pulse was 120; and there were sighing and interrupted breathing. At eleven o'clock she was more comfortable,—the pulse was 100; there was less pain of the head, and of intolerance of light and sound, less sighing, and less faintishness; she had been able to sleep for ten or fifteen minutes without feeling overcome; there was some fluttering.

"From this day the amendment was progressive, though slow. 234.

We must next present our readers with the practice to be pursued on each of these cases. This leads first to draw their attention to a plan of blood-letting, proposed by Dr. Hall, with the view of determining the *quantity* of blood which may, and should, be taken; and, in the second place, of determining from this the *diagnosis* of the disease. The plan is this. The patient is to be placed upright, and bled to incipient syncope. If there be inflammation, much blood will flow before syncope occurs, and much blood ought to be taken; in intestinal irritation, on the contrary, and, à fortiori, in exhaustion, the abstraction of a very little blood will induce syncope. Thus the taking too much, and too little, blood is guarded against;—and it is difficult to say which is the more fatal error of the two. No doubt this plan will admit of modifications—of exceptions. "Nulla perpetua præcepta medicina recipit." We shall here introduce an extract or two from Dr. Hall's methodus medendi.

"I now proceed to state the treatment of puerperal inflammation.

"And I would observe, in the first place, that nothing can be trusted to, to save the patient, but the most ample blood-letting, and, in the second place, that nothing should preclude the use of this remedy but the actual existence of the state of sinking. In regard to the measure, and the repetition of the blood-letting, many points must be taken into consideration. The earlier, and the more fully, this remedy is employed, the more efficacious and the safer it is, and the safer is its full repetition.

"There is one point which I would particularly impress upon my reader. It is, that the blood-letting should, in this disease, ever be performed, the patient being in the erect position; and it may then, in general, be safely carried to deliquium. I do not recommend this mode of proceeding with the view of producing deliquium merely; but also, that this deliquium may serve us as a guide, in judging of the extent to which we may carry the depletion. If the patient be sitting upright, and faint by the loss of blood, we have a security and remedy against any danger from this event, in laying the patient low. But if deliquium be induced by bleeding the patient in the recumbent position, I cannot say that I think it will always be without danger. I think the plan which I have proposed at once far more safe, as well as far more efficacious in subduing this disease. If it were requisite, the patient's head might be laid even lower than the rest of her body.

"The same rule may apply for the repetition of the blood-letting. If the fullest effect is desired which the patient can safely bear, let her be bled to syncope in the erect posture. She will faint from losing a larger, or a smaller, quantity of blood, precisely in the inverse proportion of the previous exhaustion; the state of syncope will not only warn us to desist from drawing more blood, but will arrest the flow of blood itself, just at the point when the patient can bear to lose no more.

"This is a most important criterion for the employment of a most powerful remedy. I do not by any means wish it to be understood, that it is always safe to bleed to deliquium in the erect posture; but that, when it is determined to bleed, it is important to have the boundary, which it would be unsafe to pass, at least clearly defined. Sometimes the patient will faint on being merely placed upright; is it then, ever, and in what particular cases, safe to bleed?

"The next question is in regard to topical blood-letting. And I think there is one important rule for the adoption of this remedy. It may, of course, be enjoined to be done immediately after general blood-letting. But it is particularly useful in those cases, in which the system is obviously subdued by the general blood-letting, and yet the inflamed part remains tender under pressure. In such cases, leeches, or still better, cupping, if it be properly and tenderly performed, will prove a most useful remedy.

"It is quite unnecessary to state the utility, or rather the necessity, for the administration of purgative medicines in this disease. There is good reason to suppose that some cases have been subdued even by this remedy alone. And the efficacy of purging in conjunction with blood-letting is quite undoubted. A constant catharsis should be kept up, indeed, until the disease is completely subdued." 190.

### INTESTINAL IRRITATION.

The treatment of this puzzling state is as follows:—

"In the treatment of the effects of intestinal irritation, I would by no means exclude the use of the lancet. Blood-letting may be useful in such a case, for the same reason that it is useful in simple fever. But I would repeat, that this remedy is only subsidiary to the full and free evacuation of the bowels, and, if necessary, of the stomach. If it were trusted to alone, or with only a moderate attention to the state of the alimentary canal, or if it were used in the manner which is required to be efficient in puerperal inflammation, I am persuaded that the patient would die of exhaustion, before the symptoms would yield.

"The remedies of intestinal irritation and its effects, I would enumerate and arrange in the following order: first, the full evacuation of the intestinal canal; secondly, blood-letting; thirdly, some kindly anodyne; fourthly, leeches, cupping, a lotion, a liniment, or a blister, according to the circumstances of the case, for the topical affection; fifthly, the mildest, nutritious food; sixthly, the most absolute quiet, and the most perfect security from light, noise, disturbance, and every other source of excitation; seventhly, every soothing plan; eighthly, great coolness, and free ventilation of the sick-room; and, lastly, a constant watching over the patient during sleep, to avoid the injurious effects of turbulent dreams on one hand, and of too long sleep and fasting on the other. Upon each of these points I proceed to make such observations as I have learnt, from practice, to be of importance.

"In regard to the state of the alimentary canal, it is quite obvious that an emetic is the proper remedy when the symptoms can be attributed to any indigestible substance taken. And I would recommend this remedy, even although it might appear, from the lapse of time, unlikely that the injurious substance should still remain in the stomach.

"When the case originates from intestinal irritation, I would earnestly recommend that the first remedy should be an enema, consisting of three or four pints of warm water, very slowly and gently forced into the bowels. This should be followed by an active purge. And this should, in due time, be followed by a repetition of the injection. I need scarcely observe, that the evacuations should be immediately carefully examined, and the effects upon the symptoms of the disease be watched.

"To abate the general heat and excitement of the system, to relieve the head or the abdomen, and to ensure perfect safety, the patient should, in cases in which the strength is not particularly impaired, be raised into the erect posture, and be blooded until faintishness be induced. This effect also should be carefully watched and observed. If it occur from the loss of a small quantity of blood, it confirms the diagnosis; if it do not occur until much blood have flowed, it should suggest the suspicion of more than mere intestinal irritation,—of one of those mixed cases which so frequently occur, and of which I propose to treat in a subsequent chapter.

"I do not imagine that this decided use of the lancet can ever be attended with danger, if there have been no previous loss of blood, or other cause of exhaustion. But it could not be repeated with impunity. It would lead to exhaustion with the symptoms of re-action, to the state of sinking, or even to sudden dissolution. And if the case be really one of intestinal irritation, and the other remedies have been duly applied, such repetition of blood-letting will not be required.

"It is an observation of great importance, that, in inflammation, repeated blood-letting is required, and is borne with safety; in intestinal irritation, on the contrary, the repetition of blood-letting is neither necessary nor safe.

"This free evacuation of the bowels, and detraction of blood, are very apt to be followed by symptoms of hurry and alarm in the system. These effects are frequently prevented by the timely administration of an efficient and kindly anodyne; and I believe no anodyne is possessed of these qualities in a higher degree than the liquor opii sedativus of Battley. Of this excellent medicine a full dose may be given, and, if necessary, repeated in five or six hours." 221.

We now come to the 7th chapter, on "mixed cases." These, according to Dr. Hall, are those which are most commonly met with in practice, and he proceeds to illustrate the various combinations of inflammation with intestinal irritation—or of either or both, with the effects of loss of blood, by a critique on the work of the late Mr. Hey, of Leeds, who, as well indeed as almost every other writer on this subject, appears to Dr. Hall to have combined in one description, all the three different states of which he (Dr. H.) has treated. We are unable to follow Dr. Hall through this critique; but we cannot help fearing that there is but too much justice in his strictures. We have, ourselves, seen so much mischief done by mistaking irritation for inflammation, that we cannot doubt of the great extent of that mischief in former times, when bleeding was so unsparingly as well as indiscriminately employed.

We shall extract the following passage on puerperal mania.

"There is a mixed case which shows itself under a still different form, from any which have hitherto been described:—it is *puerperal mania.* I believe this disease to result, in general, from all the circumstances following parturition combined; but chiefly from the united influences of intestinal irritation and loss of blood. I purpose to pursue this subject hereafter. In the mean time, however, I would observe, that I am persuaded that real puerperal phrenitis is comparatively a rare disease,—that puerperal mania is seldom of an inflammatory character, and that it is, especially, to be treated by those measures which are suited to the mixed case of intestinal irritation and exhaustion. This opinion is confirmed by the fact of mania occurring from undue lactation, as well as from the circumstances of the puerperal state. I am inclined to attribute much more to the combined influence of irritation and exhaustion, than to the mere 'state of the sexual system which occurs after delivery,' which has been assigned as the chief cause of this morbid affection by Dr. Gooch, in a most interesting paper upon this subject, in the sixth volume of the Transactions of the College of Physicians, p. 280,—although I would by no means exclude the influence of this principle altogether. There is ample evidence, in Dr. Gooch's cases, of the influence of intestinal disorder; and the events of labour, and the circumstances of lactation, ever add to this a state of exhaustion. This view is the more important, because it directly suggests the proper mode of treatment, which consists in restoring the system to a state of due health by every means in our power, whilst we adopt every measure which can soothe and allay the morbid irritability of the nervous system.

"I am confirmed in this view of the nature of puerperal mania, not only by a careful investigation of its causes, and the good effects of the remedies which I have mentioned, but by having met with the symptoms of intestinal irritation described in chapter V., as a prelude to those of mania." 253.

These remarks are illustrated by a very interesting case, some few particulars of which we shall lay before our readers.

A lady was confined after a very tedious and painful labour, and continued to do well for 50 hours, when she became affected with severe pain and beating in her head, attended with intolerance of light and sound. To these symptoms succeeded delirium, with a pulse of 130 to 140, starting and alarms, frightful visions, &c. She had no rest or sleep for five days. Leeches, purgatives, and anodyne draughts did no good, and the symptoms daily increased in violence. A drachm of the tinctura opii with spir. ammoniæ aromat. was given and repeated in the night, snow being applied to the head. This induced a profound and quiet sleep, and the patient awoke free from delirium. The same medicine was exhibited the succeeding night, and, from that time all the bad symptoms subsided.

The remainder of the chapter, being critical of Mr. Hey's work, we shall pass over.

In the 8th chapter, Dr. Hall details some cases illustrating the fatal effects of blood-letting in puerperal affections.

"These cases illustrate several points of great practical importance: and first, the danger of the repetition of the blood-letting, in cases which have been relieved by previous remedies, as a preventive merely; in such cases, all inflammation, if it existed, having subsided, a chief source of safety in the use of the lancet, as well as of the necessity for it, is removed, and the patient will be very apt to fall a prey to the further loss of blood. This is exemplified in the first and second cases about to be adduced. In the second place, I consider the particular danger of an unguarded use of the lancet, in cases not inflammatory, to be exemplified in the third case, which was clearly one of intestinal irritation, and not of inflammation. The last case is a sad instance of an inconsiderate blood-letting, and it is to be hoped that few such examples have occurred, although, I confess, that in the prevailing mania for blood-letting, even such cases should not greatly surprise us." 287.

We shall endeavour to make room for one of the cases here detailed.

"Mrs. ——, aged 33, weakly, was confined of her sixth child, after an easy labour, without flooding, at midnight on the 20th July, 1818. During the ensuing day all was well. The lochia were natural; there was no alvine evacuation, but the bowels had been open during pregnancy, and twice evacuated during labour.

"On the morning of the 22d, Mrs. —— took half an ounce of the oleum ricini; and at four in the afternoon this medicine was repeated, the first dose having produced no effect; this, however, induced violent purging, occasioned great fatigue, and caused the patient to complain much. At ten o'clock in the evening, Mrs. —— was seized with rigor, which was violent and continued more than an hour; this was followed by great heat of skin, with wakefulness, restlessness, anxiety, sighing, and moaning.

"At ten on the succeeding morning there were great heat of skin, and pain at the bottom of the back. Four teacupfuls of blood were taken from the arm.

The symptoms still continued, and at seven in the evening, three teacupfuls of blood, and at eleven three more, were taken from the arm, and twenty leeches were applied to the region of the uterus for the increased pain. The pain still continued to increase, with restlessness, sighing, faintishness, constant necessity for the smelling bottle, and apprehension of impending dissolution.

"Afterwards the symptoms being unabated, a physician was consulted.—About three o'clock, three teacupfuls of blood were again taken from the arm, and leeches again ordered to be applied; an enema was given, which evacuated a quantity of fæces quite unexpected.—In a short time Mrs. ——— became cold, and the surface clammy, with fainting, gasping, breathing, &c. and all was done to restore warmth. After an interval of three hours the pain was still great. Some opening medicine was prescribed. But the state of sinking continued,—the smelling bottle, the fan, and fresh air were urgently called for. All the symptoms, except the pain, were aggravated, there were gasping, a slight convulsive struggle, another, and the patient expired.

"In this case it will be observed, that the pain remained unabated, even after the last fatal blood-letting. I have reason to regard this, as denoting not an inflammatory origin of the pain, but the presence of morbid alvine contents." 294.

The ninth and tenth chapters on epidemic puerperal fever, and on the effects of previous disorder on the general health, need not detain us, as they occupy only a few pages of the original work—reference being made to Dr. Hall's paper in the 13th volume of the Medico-Chirurgical Transactions, of which we have already given an account in this Journal.

With these extracts and observations we must take leave of Dr. Hall for the present, and proceed to the work of Dr. Gooch.

The first remark made by this author is one in which we fear we cannot concur, viz. that the designation of peritoneal fever is to be preferred to that of puerperal fever or puerperal peritonitis. Dr. Gooch prefers this term, because "it would express the fact, that an affection of the peritoneum is an essential accompaniment of the disease, without defining what that affection is, because it is not uniform." 2. But if there be sometimes traces of peritonitis on examination, and sometimes not, to which we suppose this last remark refers, should not the two cases be distinguished by a distinct nomenclature altogether?

Leaving this point, we proceed to state that this disease is remarked to be more prevalent at one period that at another—that, when most prevalent, it is also most dangerous—that when thus prevalent, "it is not uncommon for the greater number of cases to occur in the practice of one man, whilst the other practitioners of the neighbourhood, who are not more skilful or more busy, meet with few or none." 4.

Dr. Gooch observes—

"The most fatal disease to which lying-in women are subject is known under the names of Puerperal or Child-bed Fever, Puerperal Peritonitis. Its essential symptoms are pain and tenderness over the abdomen, with a rapid pulse.* It

* "'Dr. Lowder adopted a very good method to form an accurate definition of this disease. He read all the different authors of character who had written on the subject, and noted down all those pathognomonic symptoms which they agreed were necessary to constitute the disease, and, on comparing these with his own experience, he found them to be very few—fever, intense pain of the head, and intense pain of the abdomen.'"—(Lowder, MS. Lectures.)

begins a few days after delivery, with pain of the abdomen, shivering succeeded by heat, and a quick pulse. As the disease advances, the milk becomes suppressed, the belly tumid, and the breathing short; when it terminates fatally, it does so commonly about the fifth day, but often in less than half that time. On opening the abdomen, the morbid appearances are not uniform, but the most common and remarkable are a copious effusion of lymph and serum on the surface and in the cavity of the peritoneum. Thus it is a fever essentially complicated, with an affection of the peritoneum." 2.

"From the little I have already said about the symptoms of this disease, and the morbid appearances discovered after death, it is clear that it essentially consists in fever, with an inflamed state of the peritoneum; but fever may vary, not only in degree, but in kind, or (as it is commonly called) type; and inflammation may vary, not only in degree, but also in kind or type. Hence, in investigating the nature of this disease, one of the first questions is, whether it is strictly uniform, differing only in degree in different cases, and requiring only different degrees of the same treatment; or whether it differs so much in kind or type, that the mode of treatment which is necessary in some cases, is destructive in others. The latter is the conclusion to which we must inevitably come, at first sight, in tracing the history of this disease in the works of those who have written about it. This I propose to do through the last half century, not for the useless purpose of raking up old and obsolete opinions, but because it is unsafe to draw inferences, except from a wider survey of facts than the experience of a single individual, or of a single epidemic, affords." 6.

Dr. Gooch proceeds to review the opinions of Dr. Butter, Dr. John Clarke, Dr. William Hunter, Richter, Dr. Lowder, Dr. Denman, and Dr. Gordon, and concludes by the following remark :—

"From the foregoing account of the experience and opinions of different physicians, who had opportunities of observing puerperal fever, what inferences are we to draw? Supposing that each observed accurately the disease which he witnessed, and that no mistake was made in the formation of his opinion, the inevitable conclusion is this:—that puerperal fever, by which I always mean that fever which is accompanied by an inflammatory state of the peritoneum, is not one uniform disease, but may occur under different forms,—that sometimes it is so mild as to be curable by the gentlest aperients, and at other times it is very obstinate and fatal. That in this latter form it sometimes consists of acute inflammation of the peritoneum with inflammatory fever which bears, and is curable only by early and active depletion, sometimes of inflammation and fever of a low type, in which depletion is useless, and even pernicious." 16.

Dr. Gooch next details the experience of M. Doulcet of Paris, and Dr. Böer of Vienna. Lastly, our author notices the more recent works of Dr. Armstrong and Mr. Hey, and concludes with the following remarks :—

"The Treatises of Dr. Armstrong and Mr. Hey produced a strong impression on the minds of medical men in this country. It convinced them that the puerperal fever which was then, and had been for several years, infesting various parts of the island, was an acute inflammation of the peritoneum, and that bleeding and purging, employed very early and very actively, was the only mode of treatment which was capable of arresting it: but the impression did not stop here; it produced a general conviction, that the present was a fair representative of all former and all future puerperal fevers; that bleeding and purging were its essential remedies in all places and seasons; that they had failed only because they had been used too late and too sparingly, and would succeed if they were used early and actively. In short, that a light had been thrown on the nature of puerperal fever, which explained the failures of physicians in times past, and would ensure them success in times to come. Of these conclusions, as far as they relate to the disease which the writers had been witnessing and treating, I have no doubt; but as far as they relate to past and future epidemics, it may be useful to examine the reasoning which led to these conclusions, and how far they have been corroborated by subsequent experience.

"I have no doubt, from the symptoms and progress of the disease which Mr. Hey witnessed at Leeds, and Dr. Armstrong at Sunderland, that it was a genuine puerperal fever; that is, fever accompanied by an affection of the peritoneum, although the proof by dissection was wanting in both.* I applaud the zeal and ability with which they investigated its nature, and conducted its treatment; but a question of great difficulty and importance still remains, namely, whether the puerperal fever, accompanied by an affection of the peritoneum, and often epidemic, does not assume different types in different seasons, being sometimes acutely inflammatory, and bearing and requiring early and active depletion; at others, characterized by debility, or what has been called action without power, in which depletion, however early and actively employed, is useless and pernicious." 33.

Dr. G. observes, that there are only two sources of materials for solving this question—a comparison of past epidemics with those which fall under our own observation—and an application to future epidemics of the treatment which was found so successful in those we have witnessed. The *latter* only affords conclusive proof. When Dr. Armstrong and Mr. Hey published, they were in possession only of the *former* source of information—"and they appear to me to have laid far two much stress upon it, and permitted it to lead them to far too positive conclusions." Dr. Armstrong had remarked that "there is perhaps no disease more uniform than puerperal fever in the symptoms and morbid derangements which it induces." Dr. A. also quotes Dr. Hulme as to the "immutability of the puerperal fever." On these passages Dr. Gooch makes the following comment:—

"In the leading circumstances of the disease, there is certainly great uniformity; it almost always commences a few days after delivery, is marked by pain and tenderness of the belly, and a rapid pulse; if not cured, terminates fatally within a week, and, after death, commonly leaves the depositions and effusions of inflammation. Thus far it is very uniform, but no further. To say nothing of its causes, there are at least three things requisite to form the history of a disease:—1st. Its symptoms. 2d. The effects produced by remedies. 3d. The morbid appearances discovered after death. In the history of puerperal fever there is, even in the first and third of these particulars, considerable difference: this is apparent even in the experience of Dr. Armstrong himself, who describes the local inflammatory symptoms as being sometimes very distinct, sometimes very indistinct, and sometimes absent altogether, the patients not only complaining of no pain in the abdomen, but bearing pressure without the slightest shrinking. Compare too, the symptoms of the disease as described by Dr. Gordon, of Aberdeen, and those described by Dr. Clarke, of London. In the appearances discovered after death, there is also this great difference in different epidemics, that sometimes there are the effusions of inflammation, with extensive redness of the peritoneum, at other times the peritoneum is quite pale; in the sequel it will appear that there is a still greater difference in the appearances after death. But if from the symptoms and morbid appearances we pass

---

* "'It is to be regretted that no examination could be obtained, as morbid dissection might perhaps have thrown some additional light on the nature of the disease.'—*Armstrong on Puerperal Fever, p.* 10. Mr. Hey also makes a similar complaint."

to the second particular in the history of the disease, namely, the effects of remedies (which form not only an essential, but the most important part of this history, for the two others are of no value but as they throw light on this,) there is perhaps no disease of which the histories have been so opposite. Richter could almost always cure it. Dr. William Hunter and Dr. Clarke could scarcely ever cure it. In Dr. Lowder's time it was observed that every woman who was blooded died. In Dr. Armstrong's time it was observed that every woman who was not blooded died." 36.

Throughout the whole chapter on the pathology of the disease, Dr. Armstrong appears to our author to write as if he thought that the symptoms during life and the appearances after death, were infallible guides to the nature and treatment of the disease.

"Thus, he remarks, that if a practitioner were to see a woman soon after delivery suffering pain in the abdomen, a quick pulse, and the other signs of fever, and after death were to find no other morbid appearance than extensive traces of inflammation in the abdomen, he would at once conclude that the disease was active inflammation, and would in future treat it as such; and, alluding to those writers who have considered the low epidemic fever of child-bed as a different disease from peritoneal inflammation, he says, 'it becomes a matter of great practical consequence whether symptoms and dissections justify such a distinction.' It appears to me that symptoms and dissections cannot settle the question, and that Dr. Armstrong lays more stress on the argument than it will bear. Supposing many cases of a disease, which bore in general a striking resemblance to one another in the symptoms and the appearance on dissection; this would naturally suggest as a strong probability that they would all be affected in the same way by the same remedies: but suppose that on applying these remedies to all these cases, with the same activity, at the same stage of the disease, and as far as can be made out, under the same circumstances, they produced different effects on different cases, some being relieved and recovering, others being made worse and dying; this would be more conclusive evidence of a difference between these cases, than the symptoms and morbid appearances were of their identity. The effects of remedies on a disease, if accurately observed, form the most important part of its history; they are like chemical tests, frequently detecting important differences in objects which previously appeared exactly similar. How many diseases are there in which the symptoms are inadequate guides; in cases apparently syphilitic and apparently similar, some as soon as mercury affects the mouth begin to mend, and rapidly recover; in others, the ulcers begin to spread; and so imperfect are the appearances as guides, that I have known the first surgeons in the profession giving opposite opinions about the same case, and a nose lost from taking the opinion of the majority. The local pains and constitutional disturbance which occur in feeble and bloodless persons, and which are aggravated by bleeding and other evacuants, strikingly resemble the local pains and constitutional disturbance which occur in vigorous and plethoric persons, and which the lancet and other evacuants relieve and ultimately cure; yet how many years is it before the young practitioner learns that there are cases apparently so similar yet really so different, and how to distinguish them,—and how many practitioners are there who never learn it at all! Symptoms and dissections can never do more than suggest probabilities about the nature of a disease and the effects of a remedy on it. A trial of the remedies themselves is the only conclusive proof. Sydenham was so aware of this, that he says, 'Epidemic diseases may seem alike to the unwary, because in some sort they do agree to outward appearance;' adding this confession, 'when a new species of fever arose I was doubtful how to proceed, and notwithstanding the utmost caution could scarce ever preserve one or two of the first patients from danger,' so far from infallible were symptoms as guides." 39.

Having thus discussed the history of former epidemics and the opinions of former writers, Dr. Gooch, proceeds to detail his own experience. The chief results of this were, from what obtained between the years 1812 and 1820, that puerperal fever was a fever attended by acute inflammation of the peritoneum; that the inflammatory stage was often very short, soon terminating in great and irremediable effusion into the peritoneum; that the disease was curable only in the inflammatory stage by active bleeding and purging, and that, although it was impossible to draw the line, and say when the inflammatory stage terminated in that of effusion, because it differed in length in different cases, yet that it was often incredibly short, and that the treatment had not a chance of success unless begun during the early hours of the disease. These results were precisely similar to those obtained by Dr. Armstrong and Mr. Hey. We do not therefore consider it necessary to detail them.

Dr. Gooch proceeds to the detail of several cases of puerperal disease which are of a very different character from those hitherto mentioned. They appear not to be inflammatory, not to bear or require blood-letting, but to yield to opiates, and to poultices to the abdomen, and, if fatal, not to leave traces of disease over the surface of the peritoneum. The first cases given are of a sporadic character. They are followed by others which appeared epidemically. We shall quote them in the order in which they presented themselves to the author, and in which he has transmitted them to us.

"For some years I supposed that the group of symptoms which indicate puerperal fever, always indicated the same disease, differing only in degree, so that if a woman a few days after delivery had diffused pain and tenderness over the abdomen, with a rapid pulse, she must necessarily have a peritoneal fever, for which the only remedy was early and active depletion. This, I know, was the common opinion of the profession, and may be the common opinion now, although some practitioners may have arrived at a different conclusion. The first case which led me to suspect that there were exceptions to this rule, and afforded me some light on this difficult and important subject, was the following:

"*Case* 1. The patient was a lady, twenty-six years of age, habitually thin, and long subject to occasional pains in the pelvis, which she called spasms of the womb, and which she relieved by opiates. She also frequently fell into a state of debility from which she was always restored by steel medicines, or chalybeate waters. This lady was delivered of her third child, after a short, easy, and natural labour. On the second morning after her delivery being perfectly easy, and complaining of nothing, she took a purgative, as is usual, of salts and senna. It operated plentifully several times; then the stools became frequent and watery with severe griping, and gradually the abdomen became painful and tender all over; the pain was without intermission, and the tenderness so great, that she could not bear the slightest pressure; nevertheless her skin was cool, and her pulse continued soft and not more than 80. This case occurred at a time when it was the prevailing notion in the profession, that puerperal fever was invariably acute inflammation of the peritoneum, for which early, full and repeated bleedings form the essential remedy, and I found it very difficult to prevent this treatment being adopted in this case. The medical attendant of the family visited her several times a day, insisting that the pain arose from inflammation of the peritoneum, and urging the necessity of bleeding. I succeeded, however, in preventing this measure; the abdomen was kept constantly covered by a bag of scalded bran; she took ten grains of compound powder of ipecacuanha, every four hours; the pain soon diminished, and at length subsided; the tenderness remained longer, but ceased on the second day, leaving her quite free from any symptom of disease. A few days afterwards, the bowels being confined, and mild aperients not acting, she took another dose of salts and senna, which operated as the former, producing another attack of pain and tenderness of the abdomen, which was speedily relieved by fomentations and opium." 65.

In this case, Dr. G. observes, there was not much danger of going wrong —the slow pulse was a sufficient guide.

"Without stirring the question, whether the irritation excited by the purgative had excited the symptoms which I have described, the case taught me that a lying-in woman might have a diffused and permanent pain over the abdomen with tenderness, which neither I nor the family apothecary could distinguish from the pain and tenderness of peritoneal inflammation, which nevertheless did not depend on inflammation; at least it was unaccompanied by quickness of pulse, and it was cured without depletion, by fomentations and opium. All this was new to me, and interested me much; but I had not been brooding over it long, before I met with the following still more remarkable case." 66.

*Case* 2. This was the wife of a surgeon in the country, to whom our author was called. She had been delivered a few days, and was represented to be in great danger from peritoneal inflammation. The following particulars were collected on Dr. G.'s arrival.

"In her ordinary health she was pale, and subject to fits, either hysterical or epileptic; she had been confined four days, and her present symptoms had lasted rather more than one; they were permanent pain all over the abdomen, tenderness, and soreness, so that turning in bed was distressing to her; she had had no rigors nor chilliness; her pulse was 116, perfectly soft, and rather languid. One of the medical gentlemen, who had seen her at four o'clock that morning, had taken from the arm two cups of blood; but although it had flowed in a full stream, the surface of the crassamentum was flat and red, and the bleeding had afforded her no relief. I ordered the abdomen to be covered by a linen bag stuffed with hot bread and water poultice, and I gave her twenty minims of laudanum; the poultice was to be renewed often enough to keep up heat; she was to continue the laudanum in doses of ten minims every four hours, and take nothing for diet but thin hot gruel. I breakfasted in the house; before leaving it, I went up to her chamber, and found that her abdomen was easier, her skin moist, and her pulse had fallen to 100. The next day, I received a note from her husband, to tell me that the pain and tenderness of the abdomen were quite gone, and that her pulse had fallen to 90. She now left off her opiates, but continued her fomentations, took a mild aperient, and recovered without any further interruption." 67.

In this last case there was more danger of going wrong than in the former. The pulse was quick—and there was a group of symptoms that are generally supposed to indicate peritoneal inflammation, when they attack a woman a few days after parturition. But our author was guided by the habitual constitution of the patient—by the softness of the pulse—the uninflamed appearance of the blood—and the non-relief from venesection. This case taught Dr. Gooch, that a lying-in woman might have diffused and permanent pain with tenderness of the abdomen, and rapid pulse, and all these from a state which does not require bleeding—is not relieved by that measure—but which is speedily relieved by fomentations and opiates.

*Case* 3. Dr. G. went to a lady in the country who had had violent hæ-

morrhage after delivery. She rallied from this, and Dr. G. returned to town. On the fourth day the doctor was again summoned.

"When I arrived I found the same medical men in the house, who told me that she had that morning been suddenly attacked with a severe rigor, and violent pain in the abdomen; that when the rigor went off the skin became hot, and the pulse rapid; that the belly was painful, and very tender. They lost no time, but opened a vein in the arm, and took away, they told me, thirty ounces of blood, on which she became very faint, and still continued so. When I went up to her chamber, I found her with sharp features, cold and clammy on her forehead and cheeks, with a pulse scarcely to be felt; the blood was not buffed. She died before I left the house." 70.

The body was not opened, but our author shrewdly conjectures that if, instead of blood-letting, opium and fomentations had been used, the result would have been very different.

Thus far his knowledge of these cases was confined to the symptoms during life, and to the influence of remedies. It was not long before he had an opportunity of seeing the post-mortem appearances.

*Case* 4. "A practitioner sent for me to see a patient, of whom he gave me the following account: She was habitually delicate, and subject to hysteria. After an easy labour of her eighth child, her after-pains had been long and severe, but her pulse was not quick. At six in the evening of the second day it was soft, and under 80. At four o'clock the next morning, the practitioner was called out of his bed, and found her complaining of great pain and tenderness over the whole abdomen. She had been vomiting; her pulse was quick, but small and weak, and her skin temperate. He immediately bled her, letting the blood flow till she fainted. He next gave her five grains of calomel, and soon afterwards a dose of salts and senna, but the latter was vomited. Two hours after the first bleeding, the pain not having been relieved, he bled her again to fainting. Twelve leeches were applied to the abdomen, and a pill was given her containing three grains of opium. Having received this account, I went into the chamber of the patient. Her face was ghastly; it was difficult to keep her out of a fainting fit; her skin was cold and clammy, and her pulse so quick, small, and fluttering, it could not be counted. I took off the leeches, and endeavoured to revive her by warmth and cordials, but she died in the evening, about six hours after my visit, and about thirty from the beginning of pain. The body was opened the next day. *The peritoneum was healthy, but pale: there were between one and two ounces of colourless serum in its cavity; the abdominal viscera were all healthy, but pale; the uterus was contracted in the ordinary degree.*" 71.

Our author feels no doubt that, had the belly been covered with a perpetual fomentation, and an opiate been given every four hours, instead of bleeding, the patient would have been quite well in a few hours. This and other cases prove that what Dr. Gooch terms an "affection of the peritoneum," an "essential accompaniment of the disease," puerperal fever, is not necessarily inflammation—unless there be a *pale* inflammation, without any other cognizable change from perfect integrity of structure!

Within a week after the occurrence of the above case, Dr. Gooch was sent for to the same locality, to see another of the same description; but the event was more fortunate, for he treated the case successfully with opium.

"These cases opened to me a new view of the subject; they taught me that a lying-in woman might have permanent pain and tenderness of the abdomen, with a rapid pulse, independent of acute inflammation of the peritoneum, or any other part; that these symptoms may depend on a state which blood-letting does not relieve, and which, if this remedy is carried as far as it requires to be carried in peritonitis, may terminate fatally; and that the most effectual remedies are opiates and fomentations. Most of the patients who were the subjects of these attacks were women who in their ordinary health were delicate and sensitive; the attack sometimes seemed to originate in violent after-pains, gradually passing into permanent pain and tenderness, resembling inflammation; or in the painful operation of an active purgative; but it could sometimes be traced to no satisfactory cause; the patient had had a common labour, and had experienced no unusual cause of debility or irritation. The pulse in all these cases although quick, was soft and feeble; this together with the previous constitution of the patient were my chief guides; when I could trace it to any irritating cause, such as a griping purge, and when blood had been already drawn without relief, and without being buffed, I saw my way still clearer. When I doubted, I applied leeches to the abdomen." 73.

The cases which Dr. Gooch has related, and others similar to them, were speedily and completely relieved by the remedies which he has mentioned. There seemed to be nothing dangerous in this form of disease, provided the nature of it was not mistaken, and improper remedies not employed; yet it so strikingly resembled peritoneal inflammation, that it was invariably taken for it by the practitioners who witnessed it, "all of whom possessed at least that average quantity of sense and knowledge on which the public must extensively depend."

But as these cases were sporadic and with considerable intervals between them, they did not make a strong impression on our author's mind.

"In the winter of the year 1824, puerperal fever was prevalent and fatal in London and its neighbourhood. I had resigned my office at the Westminster Lying-in Hospital, and did not know, or do not remember, what was going on there; but I saw this disease repeatedly in consultation, and heard of it among my medical friends. Several instances occurred of its prevalence among the patients of particular practitioners, whilst others who were equally busy met with few or none. One instance of this kind was very remarkable: a general practitioner in large midwifery practice lost so many patients from puerperal fever, that he determined to deliver no more for some time, but that his partner should attend in his place. This plan was pursued for one month, during which not a case of the disease occurred in their practice. The elder practitioner being then sufficiently recovered, returned to his practice, but the first patient he attended was attacked by the disease and died. A physician, who met him in consultation soon afterwards about a case of a different kind, and who knew nothing of his misfortune, asked him whether puerperal fever was at all prevalent in his neighbourhood, on which he burst into tears, and related the above circumstances.

"Among the cases which I saw this season in consultations, four occurred in one month in the practice of one medical man, and all of them terminated fatally.

*Case* 5. "The first case occurred in a lady, who had been delivered of her first child, after a long and severe labour, not from deformity of bones, but from rigidity of the soft parts and the bulk of the child; it occupied nearly three days. When visited the day after delivery she was found complaining of considerable pain in the abdomen, great tenderness, oppression at the præcordia, and difficult breathing. The pulse was rapid, but the most remarkable symptoms was immense distension of the abdomen, which separated the recti-muscles to the distance of two hands breadth, and protruded between them the anterior wall of the abdomen to a considerable elevation. The projection was returned into the abdomen, and supported by straps of adhesive plaster. The feebleness of the pulse, the exhausted state of the patient, prevented blood from being drawn, but a gentle purge was given. After this had operated, the pain being worse, twenty drops of laudanum were given, and repeated every two hours for several doses, but she sunk rapidly, and died on the third day after delivery. The body was opened the next day. The intestines were found enormously distended with air, but in the peritoneum there was neither redness, adhesion, nor effusion of any kind.

*Case* 6. " The second case occurred after a common labour of the fourth child. An opiate was ordered for the after-pains, and the patient was quite well when visited on the second day. On the evening of the third she was found to have a diffused pain and tenderness of the belly with a pulse of 140, and not weak. The symptoms had not lasted six hours, the bowels had been emptied by a purgative, fourteen ounces of blood were taken away immediately, and two grains of calomel, with five of compound powder of ipecacuanha, were given every four hours. A few hours afterwards, the pain and tenderness continuing, ten ounces more of blood were taken away, by which she was much relieved. The next day she was not so well, the belly was distended and uneasy, the pulse was quick and weak, and the aspect of the patient very unpromising. A blister was applied, and the calomel was continued, but she sunk rapidly and died, little more than two days from the commencement of symptoms. The body was opened the next day, but there was neither redness, nor adhesion of the peritoneum, nor effusion of any kind into its cavity.

*Case* 7. " In the third case, the symptoms began thirty-six hours after delivery. The remedies employed were a bleeding of twelve ounces, an emetic, a purgative, and the opium and calomel, with a view of affecting the constitution as soon as possible with mercury, but the rapidity of the disease baffled all these remedies, and the patient died on the third day. The body was opened the following day, and no vestiges of inflammation were found in the peritoneum.

*Case* 8. " In the last case the patient was delivered after a labour attended by no unusual circumstances. She was seen in the evening of the second day, when she appeared quite well, complained of nothing, and had a slow pulse. She continued well till seven the next morning, when she was seized with pain and tenderness of the belly, but it was not swelled. I saw her at ten o'clock, that is, three hours from the commencement of the attack. Her pallid and weakly appearance, the feebleness of her pulse, the total absence of those symptoms in which I had formerly been so successful with the lancet, and its unfavourable effects in the cases in which I had seen it employed this season, made me unwilling to employ it. But the patient's husband was anxious that some leeches should be applied ; because a few years before she had recovered from what appeared to him a similar illness, under the care of a very eminent physician, from the use of leeches ; and the aspect of the case was so decidedly unpromising, that we were unwilling to deprive him of this consolation. Twelve, therefore, were applied to the abdomen, without loss of time, and twelve more subsequently.

" On the evening of the second day of her illness, when I went into her bedchamber, I found her medical attendant at the bed-side with a decanter of wine, of which he was giving her a spoonful. She was free from pain, calm, and clear-headed, but she was pale, faint, breathless, cold, and had little pulse. Excepting only restlessness, she looked exactly like a woman dying from hæmorrhage. The cordials revived her for a few hours, but she died the next morning at four o'clock. The last lying-in patient whom her nurse had attended had died of a similar disease, equally rapid in its course." 79.

Dr. Gooch observes that, he does not state these cases to prove the inefficiency of full and early depletion, (for in two cases it was not used, and in the others not carried to any extent)—the treatment was chiefly conducted by "one of the best practitioners he had ever known," but who had already lost confidence in the lancet in the then prevailing epidemic. He produces these cases on account of the appearances on dissection, "which indicate a disease very different from that which occasions a copious effusion of lymph and serum into the peritoneum." The following case was watched attentively by the author from beginning to end.

*Case* 9. The patient was a lady, whom I had attended in several confinements, but who was this time to lie in at a short distance from town, under the care of the neighbouring surgeon. She was a plump, pale, relaxed woman, with a languid circulation, and subject to nervous attacks. She had a weak stomach, was often troubled by flatulence, at which times dry friction with the hand along the spine would produce the expulsion of a prodigious torrent of air from the mouth with an extraordinary noise; this was her state in her ordinary health. Her labour was very quick. When visited on the second evening she was quite well; *the following morning, being still well, she took the usual purgative of salts and senna ;* it operated violently and painfully, and this was followed by diffused pain and tenderness of the abdomen with a rapid pulse. She could neither turn in bed nor bear pressure on the abdomen, but her skin was not hot, and her pulse not hard.* This was the state in which I saw her about eight hours from the commencement of pain. I gave her twenty minims of Battley's laudanum, to be repeated every two hours for three doses; and I ordered the abdomen to be covered with a bag of scalded bran. I saw her in the evening at eight o'clock; the pain of the abdomen was easier, but the pulse was rapid; she could not bear to turn, or to be pressed upon; the belly was larger than in the morning, and the breathing hurried. I now explained to her husband the danger of her condition, and said that if he wished for a consultation, in justice to the patient and to the physician, it ought to be now, not later; about two hours afterwards we succeeded in procuring the attendance of an eminent and able physician. The chief question was about blood-letting; the aspect of the case was very unfavourable, and I believe the consideration that, if it terminated fatally, more regret would be felt at the neglect of blood-letting than at the employment of it, in the prevalent state of medical opinion on the subject, determined us to use it. As it was now, if ever, that blood-letting was to be useful, we determined to carry it to faintness. The gentleman who blooded her did not practise midwifery, and had no experience of such cases; but when he felt the pulse before bleeding, he remarked that though quick (it was nearly 130) it was soft. The blood spouted from the arm during the filling of the first two cups, and the fifth cup was more than half full before she felt faint; twelve leeches were next applied to the belly, and these were succeeded by the bag of scalded bran; she took a full opiate that night, and the following morning two drachms of sulphate of magnesia every four hours. When we met however the following day, she was much worse; her belly was tumid, her breathing short, her pulse quick and tremulous, and her mind rambled. An attempt was now made to support her with cordial diet, but she became rapidly worse, and died in the middle of the next night, less than forty-eight hours from the commencement of her symptoms. The body was not opened." 83.

---

* We certainly were not aware that salts and senna formed the *usual purgative* on the third morning after parturition. We always understood that castor oil was the nurse's prescription—and we verily believe it is a much better one than that of the Doctor. He who doubts that the *peritoneal affection*, in this case, was brought on by the "usual purgative," must be a sturdy sceptic indeed!—*Rev.*

The beneficial effects of a CONSULTATION, and of *prevalent opinion*, are rendered very conspicuous in the foregoing case! Had the "BLACK dose" (*black* indeed) not been given, the probability is, that Dr. Gooch would not have been required—and had Dr. Gooch gone by his own *unbiassed* judgment, the probability is, that the patient would now have been alive!

Dr. G. remarks that, in all these cases the striking circumstances were, the rapidity of the disease (generally less than three days) and the absence of morbid appearances in the peritoneum after death—although, during life, the whole surface of the abdomen had been painful and tender—and the pulse rapid as in puerperal fever. In our humble opinion, the striking part of the business consists in this—that there are diseases of *irritation* which resemble those of *inflammation*, and which are much more rapidly fatal, if mistaken.

Dr. Gooch next adverts to the case published by Mr. Dalrymple in Farre's journal, and re-published in our 19th number, page 201, with remarks. We there observed that Mr. Dalrymple's case "might tend to check the effusion of blood during a constitution of the atmosphere which engenders a host of diseases aping the purely inflammatory, but bearing very badly the vigorous depletion to which we have been accustomed for many years past."

In the Winter of 1827—8, peritoneal fevers were very common among the in and the out-patients of the Westminster Lying-in Hospital, of which an account was published in the 11th number of the Medical Gazette, by Mr. Hingeston.

"These cases were all attended by pain and tenderness of the belly, with a rapid pulse; the pain remitted, the skin was moist, and the pulse full and compressible. Most of them were cured, without the lancet, by keeping the abdomen covered with a large, thin, hot linseed-meal poultice, and giving ten grains of compound powder of ipecacuanha, repeated till the pain was gone. If the bowels were constipated, a purgative was previously given; if they were not so, the purgative was postponed till the pain had subsided. In one case, the dry skin and sharp pulse, indicating that the affection of the peritoneum was acutely inflammatory, twenty ounces of blood were drawn from the arm; ten grains of compound powder of ipecacuanha, with two of calomel, were given, and calomel alone was repeated every six hours; twelve leeches were applied to the abdomen, and afterwards a linseed-meal poultice. The gums became sore in twenty-four hours, and the patient recovered; but after the bleeding she had frequent faintings for several hours, and 'life was reduced to a low ebb.' A striking contrast this to the way in which bleeding to double this extent was borne in the peritoneal fevers from 1810 to 1820. Mr. Hingeston thinks that these cases, which are cured by opium, are the first stage, a lower degree of that acute inflammation which requires the lancet.

"For the following particulars I am indebted to Dr. Ferguson, one of the present physicians to the Westminster Lying-in Hospital.

"During the late autumn and winter there has been much sickness for so small a hospital. To say nothing of the out-patients, sixty-two in-patients were admitted between September 11, 1828, and February 20, 1829; of these, twenty-eight (that is, nearly half the number) had peritoneal fever; and of these, seven died, that is, one in four. A large proportion of them were cured at once by ten grains of the compound powder of ipecacuanha, every three or four hours for three doses, and a hot linseed-meal poultice over the whole belly. Sometimes this treatment removed the pain, but not the soreness; but this was generally removed by the application of leeches. As soon as the symptoms were subdued, the bowels were opened by a mild purge; when these remedies failed the case was a bad one. It may be said by the advocates for blood-letting, that these bad cases might have been saved if they had been blooded and purged early and actively; but in those in which the lancet was used, however early, its effect was discouraging. The patients fainted after losing a few ounces, the blood bore no marks of inflammation, and the remedy was followed by great and immediate exhaustion; even leeches, when applied in considerable numbers, and when they bled profusely, in some cases seemed to occasion great exhaustion. But in the cases which I have related from my own experience the lancet was used as early and actively as the warmest advocate for depletion could wish. Case No. 6 was blooded without relief, and was immediately relieved by opiates and fomentations. Cases 7 and 8 were blooded immediately on the attack of pain, the former to thirty ounces at once, the latter twice to faintness, beside leeches, calomel and senna mixture, and they immediately and speedily sunk under the remedies. Of the four cases which occurred to one practitioner, No. 10 was blooded immediately after the attack, in the evening, and the bleeding was repeated the following morning. The Case, No. 13, began during the violent operation of a purgative; the patient was blooded to faintness about fourteen hours after the attack, the blood was not buffed, and she never rose out of the exhaustion which followed. In the case related by Mr. Dalrymple the patient was blooded freely five hours from the attack, and the effects was similar." 89.

Bad health prevented Dr. Gooch from seeing many cases of peritoneal fever during the last Winter, but he has minutely detailed one, some particulars of which we shall here notice.

*Case.* A woman, previously in good health, was safely delivered at 7 o'clock in the morning of Saturday, and continued well till 2 o'clock, p.m. on Sunday, when she had a rigor succeeded by fever, and pain in the abdomen. For this she took a purgative, which operated *fifteen times*. On Monday, at 2, p.m. she had another rigor, and continued feverish. Opiates and aperients were given during Monday and Tuesday, the pain and feverishness having almost entirely subsided. In the evening of Tuesday, she was feverish, restless, with head-ache, for which leeches were applied—and afterwards she was bled to six ounces. There was no pain, at this time, in belly or back. Early on Wednesday morning the medical attendant was called up, and found she had passed a restless night, and there were now pain and tenderness of abdomen, and still more in the region of the sacrum, where the bones of the pelvis had been separated in a former labour.

"As the pulse was 120, and hard, fourteen ounces of blood were taken from the arm, and I saw her for the first time this day at one o'clock. The pain and tenderness of the abdomen were still so severe, especially in the right iliac region, that she could not breathe without stopping and crying out; and turning from her side to her back was so painful, partly from the tenderness of the belly, and partly from the pain of the sacrum, that it occupied a minute or two; the uterus was large, and could not bear pressure; above the umbilicus the abdomen was soft, and without tension; the breasts contained no milk, the lochia were scanty, the pulse, which before the bleeding in the morning had been hard, was soft, and 120; the bowels had been freely purged, and had acted that morning. The treatment agreed upon now was as follows: Twelve leeches were to be applied to the painful part of the abdomen; it was then to be kept constantly covered with a linseed-meal poultice, and when the bites ceased to bleed, fourteen more leeches were applied, succeeded by the poultice. She took twenty minims of the sedative solution of opium immediately; she was to take two grains of calomel every three hours, and five grains of the compound powder of ipecacuanha every six hours, that is with every other dose of the calomel. When we met the next day (Thursday) every symptom was better excepting the pulse; the pain and tenderness of the abdomen were gone, and she could bear firm pressure, and could breathe without inconvenience; the belly was soft and undistended; the uterus was much smaller, and the lochia copious. She was in a warm perspiration, and her breasts were full of milk, but her pulse was still at 120. As the bowels had not acted for thirty hours, the compound powder of ipecacuanha was discontinued, and a dose of salts and senna given; this was at two in the afternoon. At eleven in the evening, when her attendant visited her she was not so well: she was in no pain, but her cheek was red, she was in a great heat and perspiration, and there was a something about her aspect which he did not like. When I went the next day (Friday) to the consultation, I found the family in alarm, and was told that there had been a great change in the patient for the worse. The pain and tenderness of the abdomen had never returned, but her mind was confused; she was distressed with excessive flatulence, and the abdomen had suddenly become large and tympanitic; yet the pulse was only 116; the purgative had operated copiously, the gums were sore, and the tongue swelled. I have omitted to say that there was an inflammation about the right wrist which threatened to break. From the state of the mouth the calomel was withdrawn, and a dose of turpentine and castor oil ordered every three hours. In the evening she was still worse, bursting with flatulence, more confused in mind, and with a quicker pulse—128. She had had many little fluid motions, with a vast quantity of wind. The danger seemed so near that we made no appointment for the next day, and I never saw her again. She died that morning at seven o'clock, that is, about fifty hours from the second attack of pain of the belly.

"The body was opened the next day by Mr. Stanley. Putrefaction had made rapid progress for the time. The orifice in the vein of the arm, made four days before, had opened after her death, and discharged about half a pint of blood. On opening the abdomen the intestines were found immensely distended with air; there was no redness of peritoneum, no adhesion of its surfaces. In the lower part of the abdomen there were about three ounces of a bloody transparent fluid. In both ovaries the internal glandular structure was entirely gone, leaving only a hollow empty capsule; in that of the right ovary there was an aperture. The Fallopian tube on both sides contained pus, not at the uterine, but at the fimbriated end. There was no pus in the uterus; this organ was perfectly healthy: its veins were examined, and were undiseased; its inner surface was of a bright pink, more irregular at the part where the placenta generally adheres. The joints of the pelvis were next examined; the symphisis pubis moved readily: on cutting into it the cartilages were sound, but the space between them contained a bloody fluid. The sacro-iliac joints were still more affected; the sacrum projected from the ossa innominata half an inch; within the joint was a bloody fluid, mixed with some loose lymph." 94.

Our author suspects that the withdrawal of the opium, and the exhibition of the senna and salts contributed not a little to blast the fair prospects which dawned on the Thursday of the foregoing narrative. He further remarks that, he who looks for a copious effusion of serum and lymph as the essential appearances in puerperal fever, will be likely to deny that this was that disease at all. But Dr. G. is of a different opinion. He thinks it was puerperal fever, rendered much more dangerous than usual, by complication with disease in the sacro-iliac joints.

"The foregoing facts, and many others similar which I could relate, permit me no longer to doubt that there is a form of peritoneal fever in child-bed, which although it has the ordinary symptoms—pain and tenderness of the belly, with a rapid pulse, is very different from the peritoneal fevers which prevailed between 1810 and 1820; different in its duration, which is much shorter; in the way in which it is affected by blood-letting; and lastly, in the morbid appearances discovered after death. This form of the disease, like the acute inflammatory form, may occur only occasionally or sporadically, when it is readily and speedily cured by opiates, fomentations, gentle aperients, and sometimes leeches; or it may become prevalent or epidemic, and consequently more malignant; in other words, more fatal and difficult of cure.

"The most remarkable circumstance which the experience of the last few years has taught us about peritoneal fevers is, that they may occur in their most malignant and fatal form, and yet leave few or no vestiges in the peritoneum after death. The state of this membrane, indicated by pain and tenderness of the abdomen, with a rapid pulse, appears to be not one uniform state, but one which varies so much in different cases, that a scale might be formed of its several varieties; this scale would begin with little more than a nervous affection, often removable by soothing remedies, and, when terminating fatally, leaving no morbid appearances discoverable after death. Next above this, a state in which this nervous affection is combined with some degree of congestion, indicated in the cases which recover, by the relief afforded by leeches, and in the cases which die, by slight redness in parts of the peritoneum, and a slight effusion of serum, sometimes colourless, sometimes stained with blood. Above this might be placed those cases in which there are, in the peritoneum, the effusions of inflammation without its redness, namely, a pale peritoneum and no adhesions, lymph like a thin layer of soft custard, and a copious effusion of serum rendered turbid by soft lymph. Lastly, the vestiges of acute inflammation of the peritoneum, namely, redness of this membrane, adhesion of its contiguous surfaces, a copious effusion of serum, and large masses of lymph.

"The experience of the last few years has brought me to this conclusion: that the sanguine hopes which were entertained, a few years ago, that the peritoneal fevers of lying-in women are always of an acute inflammatory type, and always to be cured by early bleeding and purging, as they were not borne out by the reasoning employed, so they have not been confirmed by subsequent experience." 98.

How are we to act when new epidemics arise? is a question asked by our author. We believe the answer is—we must grope our way, as Sydenham did before our time. We agree with Dr. Gooch, that it is a most important object to see the patient as early as possible in the disease. But this does not depend on our own choice, but on that of nurses—"an intractable race." The next advice is—

"To undertake the treatment of these diseases with the belief that they depend not on one and the same state, acute inflammation of the peritoneum, demanding one and the same treatment, bleeding and purging, but that they depend on different states in different cases, and epidemics, which require so much caution and discrimination to distinguish, that the most cautious and discriminating practitioner will sometimes err." 100.

Lastly, Dr. Gooch would urge practitioners of midwifery to make themselves familiarly acquainted with those modes of treatment which appear, on competent authority, to have been, at times at least, most successful, so as to have them ready to apply whenever the occasion occurs. Many practitioners have but one set of ideas respecting puerperal fever, and one mode of treatment to meet it. When this fails they are bewildered, and before they can "search the scriptures" for information, many of their patients are lost.

"The remedies of the efficacy of which there is most evidence are, 1st, bleeding and purging; 2nd, emetic doses of ipecacuanha; 3rd, opiates internally, and poultices externally to the abdomen; 4th, mercury given so as to affect the constitution; 5th, oil of turpentine." 102.

On each of these remedies Dr. Gooch makes some judicious remarks. He properly observes that, there is a class of peritoneal fevers in which the affection of the peritoneum is acute inflammation—and that of the constitution is inflammatory fever—"although this inflammatory state often lasts only a few hours." These cases may be either sporadic or epidemic. We can only ascertain their nature by watching, as was said before, "the constitution of the year."

"The power of mercury as a remedy for inflammation has been so clearly made out in inflammation of the eye, the liver, and the pericardium, and not only its power, but the inadequacy of blood-letting to overcome inflammation in these organs without the aid of this remedy, that it deserves a fair trial in peritoneal fevers. I have never given it systematically in a number of cases, but what experience I have is in its favour. In the Westminster Lying-in Hospital, when ten or twenty grains of calomel used to be given every day, with purgatives, the gums sometimes were affected, and these patients invariably recovered. During the last winter it was tried in a few cases; when the gums became affected the patients recovered; but in others the disease was more rapid than the remedy, and the patients died without the mercury having affected them. The method of using it is to give two grains of calomel every two hours, until the gums become sore, or without this effect, until there is evidence that the circulation is drawn from the peritoneum to other surfaces, especially the bowels, the kidneys, and the skin; and this is attended by a subsidence of the disease, indicated by a diminution in the frequency of the pulse, a cessation of the pain, and the patient being able to turn in bed, and bear pressure. During the use of the calomel, opium generally requires to be used at longer intervals, as five or ten grains of the compound powder of ipecacuanha once in four hours." 106.

The symptoms which call for the use of opium are, severe pain, profuse purging, or sinking of the general circulation. Even when the gums are sore, Dr. G. thinks the calomel should not be suddenly withdrawn, but given at longer intervals. This is the plan successfully employed by Dr. Farre.

"There is a class of cases attended by pain and tenderness of the abdomen, with a rapid pulse, which does not require bleeding, which does not bear it to the extent to which it is necessary in the inflammatory peritoneal fevers, and which is speedily and effectually cured by opiates internally, with hot poultices to the belly, aperients, and sometimes leeches. There is reason to believe that this form of the disease is present when the patient in her ordinary health is delicate and nervous—when the pain and tenderness have followed any irritating cause, such as severe after-pains, or a griping purge—when the pulse, although quick, is perfectly soft, and even weak, and this opinion is strengthened if blood has been drawn without relief, and without the signs of inflammation on its surface. The best way of treating these cases is to wash out the large bowels by a very large glyster, to give ten grains of compound powder of ipecacuanha every three hours, till the pain is gone, to keep the abdomen constantly covered with a warm linseed-meal poultice, and after the pain has ceased, if the abdomen continues sore, and the pulse quick, to apply leeches, and give a mild purge. When I doubt the nature of the case, I apply leeches at the beginning." 107.

We have now brought the subject of peritoneal or puerperal fever to a close, and have endeavoured to let both authors speak for themselves, and, as much as possible, in their own idiom. In this respect we have somewhat deviated from our usual course, that of preferring condensed analysis to frequent extracts. Such works as those which are now before us do not often present themselves—and we have proportioned the extent of our reviews to the value which we conceive they possess. To the several other articles in Dr. Gooch's book we shall dedicate such space as we deem advisable; but with them, we shall be able to deal more in our usual analytical style.

